# Diabetes Mellitus in Kidney Transplant Recipients: New Horizons in Treatment

**DOI:** 10.3390/jcm14041048

**Published:** 2025-02-07

**Authors:** Maya Sanchez-Baya, Mónica Bolufer, Federico Vázquez, Nuria Alonso, Elisabet Massó, Javier Paul, Veronica Coll-Brito, Omar Taco, Paula Anton-Pampols, Rosana Gelpi, Iara DaSilva, Ángela Casas, Rosely Rodríguez, Maria Molina, Laura Cañas, Anna Vila, Jordi Ara, Jordi Bover

**Affiliations:** 1 Department of Nephrology, Hospital Universitario Germans Trias i Pujol, 08916 Barcelona, Spainmcollb.germanstrias@gencat.cat (V.C.-B.); mmolinago.germanstrias@gencat.cat (M.M.);; 2 Redes de Investigación Cooperativa Orientadas a Resultados en Salud (RICORS) 2040, 28029 Badalona, Spain; 3 Department of Endocrinology and Nutrition, Hospital Universitario Germans Trias i Pujol, 08916 Barcelona, Spain; 4 Germans Trias i Pujol Health Sciences Research Institute (IGTP), 08916 Badalona, Spain; 5 Department of Medicine, Autonomous University of Barcelona (UAB), 08193 Barcelona, Spain; 6 CIBER of Diabetes and Associated Metabolic Diseases, Instituto de Salud Carlos III, 08041 Barcelona, Spain

**Keywords:** post-transplant diabetes mellitus, diabetes mellitus, cardiovascular disease

## Abstract

Diabetes mellitus (DM) in kidney transplant recipients (KTR) is a risk factor for mortality, increases the risk of infections and, in the long term, can lead to graft loss due to diabetic kidney disease. A preventive approach applied to those on the waiting list could decrease the incidence of post-transplant DM (PTDM) by detecting those patients at risk, thus allowing strategies to minimize the probability of developing a New Onset Diabetes After Transplant (NODAT). On the other hand, modifications of immunosuppressive therapy may improve glucose control in patients with KTR. In recent years, two new classes of antidiabetic drugs and non-steroidal mineralocorticoid receptor antagonists have demonstrated cardiovascular and renal benefits in randomized clinical trials where the transplant population has not been represented. Because of the potential benefit expected in this population, the clinical use of glucagon-like peptide-1 receptor agonists (GLP-1RA), sodium-glucose cotransporter 2 inhibitors (SGLT2i) and finerenone is increasing in the kidney transplant setting. This review focuses on comprehensive pharmacological interventions in KTR with glucose metabolism disorders. In-depth knowledge in this area will allow prevention and identification of potential adverse effects or drug interactions in the clinical course of KTR with DM.

## 1. Introduction

Renal transplantation is the best technique for renal replacement therapy in patients with chronic kidney disease (CKD). On the one hand, DM is the most common cause of renal replacement therapy in the world [1]; and at the same time, patients with other etiologies of kidney disease can develop DM after transplantation. Post-transplant diabetes mellitus (PTDM) is defined as newly diagnosed DM in the post-transplant setting, regardless of timing or whether it was present but undetected prior to transplantation [2,3]. However, according to the International Consensus Document [2], a diagnosis of PTMD based on hyperglycemia detected in the first 45 days is discouraged (usually transient and associated with increased exposure to immunosuppressants, infections or treatment of acute rejection) [4].

In addition to the usual risk factors for type 2 diabetes mellitus (T2DM) in the general population (age, overweight/central obesity, family history of diabetes, hypertriglyceridemia, impaired basal glycemia, metabolic syndrome, hepatitis C virus infection, and black and Hispanic race [5,6,7], there are specific risk factors in the transplant population, such as immunosuppressive medications, hypomagnesemia [8], cytomegalovirus infection [9] and episodes of graft rejection [10,11]. This implies an incidence of PTDM of 24% at three years [12]. Glucocorticoids induce hyperglycemia predominantly by exacerbating insulin resistance, enhancing gluconeogenesis and lipolysis, and reducing skeletal muscle glucose uptake. Calcineurin inhibitors, mainly tacrolimus, have a direct effect on β-cell function, leading to reduction in insulin secretion [13].

The clinical importance of PTDM lies in its impact as a significant risk factor for cardiovascular disease and CKD in solid-organ transplantation [14]. The assessment of glucose tolerance status, beginning on the waiting list, is based on a continuous risk assessment and diagnostic screening applying the same criteria for HbA1c and glucose thresholds used in the general population. The guidelines advocate the utilisation of the oral glucose tolerance test (OGTT) on account of its capacity to discern early glucose alterations, its superior diagnostic sensitivity, and its robust correlation with adverse event outcomes. Nevertheless, it should be noted that the execution of this test is intricate and its employment is infrequent [3]. This strategy allows for early intervention and treatment of diabetes and prediabetes in order to preserve beta-cell function or progression to diabetes.

So far, insulin therapy has been the primary treatment for PTDM [3] and no studies to date have firmly established which noninsulin agents are safest or most efficacious in PTDM [15]. Recent management guidelines do not provide treatment algorithms, due to the lack of randomized clinical trials, but expert opinion emphasizes the benefit of using the new cardio and nephroprotective drugs in the transplanted population [3].

In this review, we will present the most relevant published evidence in the area of renal and cardioprotection in KTR with DM.

## 2. Risk Factors for Diabetes Mellitus Post Transplant

The appearance of diabetes mellitus after kidney transplantation involves the same factors as in the general population, plus other factors specific to the transplanted population. Classic risk factors include obesity, metabolic syndrome, age, family history of diabetes and being male [5,14,16,17,18]. Specifically, in the transplanted population with hepatitis C virus infection, it has been shown that the risk of developing diabetes mellitus increases, as observed in a retrospective study of 427 patients [19]. Singularly, post-transplant diabetes mellitus has been correlated with specific common single nucleotide polymorphisms in various interleukin genes, transcription factor NFATc4 and adiponectin [20,21,22].

In the specific case of the transplanted population, the use of immunosuppressive drugs with pancreatic beta cell damage and its diabetogenic characteristics is key to the development of diabetes mellitus (corticosteroids, calcineurin inhibitors and mammalian target of rapamycin inhibitors) [23]. According to the literature, the dose from which it implies an increase in insulin resistance is 10 mg of prednisone or more per day [24]. While reducing the prednisone dose from 10 mg to 5 mg decreases insulin resistance, its total abolition does not provide an additional increase in the sensitivity of this hormone according to a study carried out on 57 kidney transplant patients on a cyclosporine-based immunosuppressive regimen [25]. Indirect insulin resistance indices can be used to predict incident PTDM in KTRs [26]. According to a Cochrane review that included more than 4000 patients, out of every 100 patients treated with tacrolimus, it prevents 12 rejection episodes and two graft losses, but induces five new cases of insulin-dependent diabetes [27].

## 3. Impact on Cardiovascular Health

Patients with DM are at increased risk of developing cardiovascular disease [28]. In T2DM, the risk of developing coronary heart disease, stroke, heart failure, atrial fibrillation and peripheral arterial disease over a lifetime is two-to-four-times higher [29].

Furthermore, DM is an important risk factor for the development of CKD, which in itself is associated with the development of cardiovascular disease [28], with albuminuria being an independent predictor of the risk of kidney failure regardless of the estimated glomerular filtration rate (eGFR) [30], making it imperative to screen for cardiovascular disease and CKD in patients with DM [28].

KTRs have an increased mortality rate and this is associated with a high incidence of cardiovascular-related death after transplantation; accounting for over 50% of all deaths in this population [31,32]. The increased risk of cardiovascular disease in KTR is associated with the accumulation of atherogenic risk factors before and after transplantation, and risk factors linked with transplantation status and treatment (PTDM, graft rejection, immunosuppressive agents) and other secondary complications of established chronic kidney disease in the renal graft (oxidative stress, anemia, volume overload, inflammation and proteinuria, secondary hyperparathyroidism) [33,34].

PTDM is one of the major risk factors for cardiovascular-related death after transplantation and numerous studies have reported the negative effects of PTDM on cardiovascular disease and mortality [34,35]. In addition, other forms of impaired glucose metabolism appear to be associated with increased mortality after transplantation [3,36]. It is for this reason that the assessment of the risk of PTDM should be performed before kidney transplantation to enable early implementation of the appropriate intervention [34,37]. The oral glucose tolerance test is the preferred test to make a diagnosis of PTDM [38].

## 4. Complications and Long-Term Consequences of DM in KTR

Patients with T2DM tend to be older and comorbid. These facts decrease their likelihood to access the kidney transplant waiting list and exclude them from the possibility of receiving a transplant [39]. In this regard, the Finnish registry in 2000–2010 (n = 5419) found that the relative probability of being transplanted was 0.25 in subjects with DM versus 0.59 in non-diabetics [40].

Long-term outcomes in patients with T2DM who receive a transplant are controversial. Lower survival in KTR with DM (70% vs. 90.5% at 5 years and 55% vs. 86.7% at 10 years) was reported by Garcia-Padilla et al. in a cohort of 375 transplanted patients, 16% of whom were diabetic [41]. In a matched control study of 62 patients with DM and 62 without DM, patient survival was also found to be lower in the diabetic group (70% vs. 83% at 5 years and 54% vs. 71% at 10 years), but no significant difference was found in death-censored graft survival [42].

Data from the Australian registry of 10,714 KTR, of whom 9% had T2DM, showed that the first-year mortality rate was twice that of subjects without DM, with the highest risk of death in recipients under 40 years of age [43]. Recently, new studies have published a significant change in this direction, with a decrease in the 5-year mortality risk in subjects with DM and survival rates similar to those without DM, as well as similar 5-year survival rates for diabetic and non-diabetic KTR (88% and 93%, respectively) [40,44].

KTR with PTDM have increased rates of infection, renal graft loss and all-cause mortality [3]. A meta-analysis of 14 retrospective studies comparing 9872 patients with PTDM to 65,327 patients without DM concluded that patients who develop PTDM have a 67% increased risk of all-cause mortality and a 35% increased risk of renal graft loss [45]. Other published studies have found no association between PTDM and patient or graft survival [46].

Interestingly, in their cohort of 1757 KTR, Hussain et al. found that pre-transplant DM increased the mortality risk by up to 66%, whereas PTDM was not a risk factor of death, even after 5 years of post-transplant follow-up [47].

## 5. New Pharmacological Interventions in KTR

In subjects with T2DM, the use of sodium-glucose cotransporter 2 inhibitors (SGLT2i) and/or glucagon-like peptide 1-receptor agonists (GLP-1RA) are recommended in addition to standard therapy (antihypertensive drugs, statins, antithrombotic therapy etc.) to reduce cardiovascular risk, regardless of glycemic control. In addition, in T2DM patients with CKD, the use of finerenone (added to angiotensin-converting enzyme inhibitors (ACE-i) or angiotensin type II receptor blockers (ARBs), when residual albuminuria is present) is recommended to reduce the risk of cardiovascular disease and renal failure [28]. These drugs have demonstrated cardiovascular and renal benefits in randomized clinical trials and real world studies where the transplant population has not been represented.

### 5.1. Sodium Glucose Cotransporter-2 Inhibitors

The main mechanism of action of SGLT2i is the reduction of sodium-glucose reabsorption in the proximal convoluted tubule; producing glucosuria, natriuresis and diuresis. This reduces filtration pressure, restores glomerular filtration and decreases renal oxygen demand. SGLT2i initially decreases glomerular filtration by inhibiting sodium and glucose reabsorption in the proximal tubule, increasing sodium delivery to the macula densa and activating tubulo-glomerular feedback, which induces vasoconstriction of the afferent arteriole. This transient reduction in glomerular filtration has been shown to offer protection to the glomeruli by reducing hyperfiltration and intraglomerular pressure, thereby slowing the progression of renal structural damage. With sustained use, SGLT2i stabilizes renal function by improving glomerular hemodynamic balance, reducing albuminuria and limiting the progression of chronic kidney disease [48].

Several randomized controlled trials have demonstrated dramatic improvements in cardiorenal outcomes with SGLT2i therapy in the non-renal transplant population [49,50,51,52,53]. Currently, only one single-center prospective clinical trial of 49 patients is available in the transplant setting, in which SGLT2i versus placebo was initiated for 24 weeks after diagnosis of PTDM at least one year after transplantation. Halden’s study showed improved glycemic control and weight loss in the SGLT2i group, with no differences in adverse events, immunosuppressive drug levels or eGFR between the two groups [54]. The rest of the available literature is based on case series [55,56,57,58] and prospective studies [59,60,61,62]. The largest study carried out in the transplant population is a Spanish multicenter study that analyzed the incidence of urinary tract infections in 339 subjects; which was 10.3% at 6 months of follow-up, and the main risk factors were being a woman (OR 2.46 (1.19–5.03)) and having had a urinary tract infection in the 6 months prior to starting the drug (OR 7.90 (3.63–17.2)) [62]. Interestingly, Lin et al. found in a propensity score-matched study that SLGT2i reduced cardiovascular events in KTR with DM, particularly the incidence of acute myocardial infarction and cardiovascular death [45]. Currently, several clinical trials with SGLT2i in KTR are underway to evaluate various aspects such as efficacy, safety and cardiorenal effects (Table 1).

### 5.2. Glucagon-like Peptide Receptor Agonists

The mechanism of action of GLP-1RAs is based on the glucoregulatory actions of “incretins”; a group of endogenous hormones secreted after ingestion by the cells of the small intestine, which act on the beta cells of the pancreas, stimulating insulin secretion. GLP-1RA drugs improve glycemic control, reduce weight and cardiovascular risk and increase survival rates in patients with T2DM [75], reducing major adverse cardiac events, lowering all-cause mortality and minimizing hospitalizations due to heart failure [75,76,77,78]. A secondary analysis of the SUSTAIN 6 and LEADER trials (which included people with T2DM without prior cardiovascular disease) suggested that semaglutide and liraglutide reduced the degree of albuminuria and the slope of eGFR decline [79]. Recently, the FLOW study confirmed that semaglutide reduces cardiovascular events, and the risk of renal disease progression in patients with T2DM and CKD. Results showed a 24% reduction in a composite endpoint of incident renal failure (dialysis, transplant, or eGFR < 15 mL per minute per 1.73 m^2^), at least a 50% reduction in baseline eGFR, or death from renal or cardiovascular causes in the semaglutide group compared with the placebo group (331 vs. 410 first events; hazard ratio, 0.76; 95% confidence interval 0.66 to 0.88; *p* = 0.0003) [78].

It is important to note that the previously mentioned randomized controlled trials excluded KTR [80] A systematic review of the use of GLP-1RAs in KTR was associated with benefits in terms of glycemic control and body weight reduction, without significant effects on blood pressure control or renal function (significant reduction in urinary albumin-to-creatinine ratio (UACR), but no significant changes in eGFR); in relation to immunosuppression there were no significant changes in blood levels of tacrolimus [80,81]. In the same way, Mahzari et al. shows that semaglutide is effective and provides significant and sustained reductions in HbA1c levels in post-renal transplant patients with T2DM or PTDM, and led to a sustained reduction in weight [82]. Semaglutide was well tolerated and led to no significant side effects [81,82].

In addition, treatment with semaglutide resulted in significant reductions in body weight and BMI in patients with obesity on dialysis, with an acceptable side-effect profile comparable to that of the non-dialysis population. These data may be important in allowing access to the waiting list for patients with obesity who, in other circumstances, would not achieve the required weight reduction for surgery [83].

It has been established by Dai et al. with human islets transplanted into immunodeficient mice treated with tacrolimus or sirolimus that the use of GLP-1RA reduces in these cases the damage of the pancreatic beta cell induced by immunosuppressants [84,85].

Some clinical trials are currently underway regarding the use of GLP-1RA for the treatment of DM and obesity in renal transplantation (Table 2).

### 5.3. Finerenone

Finerenone is a highly selective non-steroidal mineralocorticoid receptor antagonist. Compared to steroidal MRAs (eplerenone, spironolactone), there is a reduction in adverse effects such as hormonal changes including gynecomastia, less risk of hyperkalemia, etc. [88]. In addition, blocking the transcription of profibrotic and pro-inflammatory genes in several cell types at the renal site (podocytes, mesangial cells, macrophages, fibroblasts, tubular cells) will result in a lower degree of lesions, inflammation and fibrosis. This will ultimately lead to lower albuminuria and slower progression of CKD observed in clinical trials [89].

Two placebo-controlled trials, FIDELIO-DKD and FIGARO-DKD, demonstrated that finerenone reduced the risk of renal failure and a reduction of the combined cardiovascular endpoint of CV death, non-fatal myocardial infarction, non-fatal stroke, or hospitalization for heart failure in patients with CKD and T2DM who were already on maximal doses of ACE-i or ARB [90,91,92]. FIDELIO-DKD demonstrated a reduction in the risk of progression of renal disease with a primary composite outcome of renal failure, a sustained decline in eGFR of at least 40%, or death from renal causes, in patients with eGFR 25–60 mL/min/1.73 m^2^ and UACR 2–5000 mg/g, or eGFR 60–75 mL/min/1.73 m^2^ with UACR of 20–5000 mg/g [92].

In T2DM patients with CKD, finerenone is therefore recommended in addition to a RAS inhibitor in patients with T2DM and eGFR > 25 mL/min/1.73 m^2^ with UACR ≥ 2 mg/g, with appropriate potassium monitoring [93].

KTR are usually excluded from randomized clinical trials of mineralocorticoid receptor antagonists because of the potential risk of interactions between the study treatment and immunosuppressive therapy (finerenone and tacrolimus are both metabolized via CYP3A4). Finerenone should not be used concomitantly with strong CYP3A4 inducers or inhibitors [94]. Currently underway is a Phase II clinical trial evaluating the effect of finerenone versus placebo KTR to determine the feasibility, tolerability, safety and efficacy of finerenone. This study includes a kidney biopsy sub-study and a functional magnetic resonance imaging sub-study [95].

## 6. Discussion

As we are currently entering a new era in the treatment of T2DM with substantial benefits in nephroprotection and cardiovascular risk reduction, we are waiting for more evidence to extend these benefits to renal transplant patients. Although the available evidence in the field of renal transplantation is based on retrospective studies, several randomized clinical trials are underway and will soon enrich future clinical guidelines. However, even more important than having access to the best drugs available is preventing the occurrence of DM in patients without impaired glycemic metabolism before transplantation.

Although SGLT2is and GLP-1RAs are not included in the clinical guidelines’ therapeutic algorithms for renal transplant patients, their use is becoming more and more common in routine clinical practice [80,81,96]. With regard to SLGT2i, the risk of urinary tract infections must be considered, although the rate is not higher than in the general population [62]. Currently, clinical trials in KTR start SGLT2i between the third and sixth months, post-transplant.

As for GLP-1RA, despite the increased risk of gastrointestinal side effects, their benefits on metabolic control and proteinuria have been demonstrated, making it reasonable to implement their use in overweight or KTR with obesity. Another interesting aspect is the use of GLP-1RA in patients with obesity to reduce their BMI and thus facilitate their inclusion on the kidney transplant waiting list [83]. The results of in vivo clinical trials on pancreatic beta cell protection in patients receiving tacrolimus/sirolimus and GLP-1RA suggest that they may have a key role in post-transplant diabetes mellitus [85,97]. New drugs will soon be available that will complement or replace the currently established drugs such as metformin, DPP4i and insulin. The utilisation of new weekly basal insulins, namely icodec and efsitora alfa, which are ultra-rapid acting analogues or closed-loop systems for automatic insulin delivery, has yet to be explored in the context of transplant recipients. New long-acting dual or triple receptor GLP-1 agonists are entering the T2DM toolbox. They target the GLP1-R along with other receptors such as the glucagon amylin receptor (AMLNR), the glucagon receptor (GCGR), the glucose-dependent insulinotropic polypeptide receptor (GIPR), the glucagon-like peptide 2 receptor (GLP2R), and the neuropeptide Y2 receptor (NPY2R). Tirzepatide, a dual GIP and GLP1 receptor agonist, has already shown promising results on renal outcomes and cardiovascular markers in T2DM. Other promising targets with drugs in development include glucokinase activators such as dorzagliatin, amylin analogs, and imeglimin. To date, none of these drugs have been tested in KTR [98].

In contrast to the previously mentioned drugs, finerenone affects the metabolism of tacrolimus, the main immunosuppressive drug used in transplantation today. Therefore, the results of the EFFEKTOR clinical trial will be of major importance in providing knowledge in this area.

The evidence for reduced cardiovascular risk and long-term mortality in KTR is currently undefined, and further studies are needed to evaluate these medications in the transplant population.

## 7. Conclusions

It is expected that the use of the new renal and cardioprotective drugs that benefit the T2DM population with CKD will also benefit KTR. SGLT2i and GLP-1RA appear to be safe when used in the transplant population, without relevant interactions with immunosuppressant levels; however, controlled studies are needed to examine long-term mortality and cardiovascular outcomes. Their results are likely to lead to changes in future kidney transplant clinical guidelines. Unfortunately, finerenone, despite its potential beneficial effects, has a more complex profile and its future use in the transplant population will depend on further studies to demonstrate its safety and benefits.

## Figures and Tables

**Table 1:** Clinical trials with SGLT2i ^1^ and kidney transplant recipients.

Title	State	Start SGLT2i (Post-KT ^2^)	Intervention	Planned End
Cardiorenal effects of SGLT2i in KTR CREST-KT [63] Duke-EEUU	Recruiting Phase 2	>1 year	Empagliflozin 10 mg vs. Placebo (n = 72)	2024
Efficacy, mechanism and safety of SGLT2i KTR INFINITI 2019 [64] Canada	Recruiting Phase 3	>6 months	Dapagliflozin 10 mg vs. Placebo matching Dapagliflozin (n = 52)	2024
The Efficacy, mechanism and safety of SGLT2i and GLP-1 combination in KTR HALLMARK [65] Canada	Recruiting Phase 2	>3 months	Dapagliflozin 10 mg vs. Semaglutide 1 mg/mL (n = 22)	2024
Can dapagliflozin preserve structure and function in KTR? [66] Norway	Recruiting Phase 4	6 weeks	Dapagliflozin 10 mg vs. placebo (n = 165)	2025
Effect of adding dapagliflozin to allograft dysfunction of KTR [67] Brazil	Recruiting Phase 4	>1 year	Dapagliflozin vs. placebo (n = 220)	2023
Empagliflozin treatment in KTR SEKTR [68] EEUU	Recruiting Phase 4	>3 months	Empagliflozin (n = 264)	2030
Effects of empagliflozin in reducing oxidative stress after kidney transplantation [69] Iran	Recruiting	Post KT ^2^	Empagliflozin vs. Insulin (n = 40)	2025
Effect of empagliflozin vs. linagliptin on glycemic outcomes, renal outcomes and body composition in KTR with DM (EmLinaRenal) [70] India	Recruiting	>3 months	Empagliflozin 25 mg vs. Linagliptin (n = 220)	2025
A RCT to assess the effect of dapagliflozin on renal and cardiovascular outcomes in patients with severe CKD. The RENAL LIFECYCLE trial [71] Australia and Europe	Enrolling by invitation Phase 3	>6 months	Dapagliflozin 10 mg vs. placebo (n = 1500)	2027
SGLT2i ^1^ in diabetic patients with renal transplantation [72] Egypt	Active, not recruiting Phase 1 and 2	>3 months	Dapagliflozin vs. placebo	2025
Efficacy and mechanisms of dapagliflozin in promoting kidney function and cardiovascular health in KTR [73]	Phase 4 Recruiting	>1 year	Dapagliflozin 10 mg vs. placebo	2028
Dapagliflozin on renal morphology and renal perfusion in patients one year after kidney transplantation [74] Germany	Recruiting Phase 4	>9 months	Dapagliflozin + standard care vs. standard care	2026

^1^ SGLT2: Sodium Glucose Cotransporter-2 inhibitors; ^2^ KT: kidney transplant.

**Table 2:** Clinical trials with GLP-1RA ^1^ in kidney transplant recipients.

Title	State	Timing of Initiation (Post KT ^2^)	Intervention	Planned End
The efficacy, mechanism & safety of SGLT2i & GLP-1 ^1^ combination in KTR HALLMARK [65] Canada	Recruiting Phase 2	>3 months	Dapagliflozin 10 mg vs. semaglutide 1 mg/mL (n = 22)	2024
Semaglutide treatment for hyperglycemia after renal transplantation (Sema-RTx) [86] Denmark	Recruiting Phase 4	10–15 days	Oral semaglutide 3 mg vs. placebo (n = 124)	2026
Obesity management for kidney TRANSPLANTation: OK-TRANSPLANT 2 [87] Canada	Recruiting Phase 4	Before KT ^2^	Semaglutide maximum tolerated dose vs. virtual weight management coaching (n = 60)	2025

^1^ GLP-1RA: Glucagon-like peptide receptor agonists; ^2^ KT: kidney transplant.
